# Critical Evaluation of Specific Efficacy of Preparations Produced According to European Pharmacopeia Monograph 2371

**DOI:** 10.3390/biomedicines10030552

**Published:** 2022-02-25

**Authors:** Annekathrin Ücker, Stephan Baumgartner, David Martin, Tim Jäger

**Affiliations:** 1 Institute of Integrative Medicine, University of Witten/Herdecke, 58455 Witten, Germany; stephan.baumgartner@uni-wh.de (S.B.); david.martin@uni-wh.de (D.M.); tim.jaeger@unibe.ch (T.J.); 2 Center for Complementary Medicine, Institute for Infection Prevention and Hospital Epidemiology, Faculty of Medicine, University of Freiburg, 79106 Freiburg, Germany; 3 Institute of Complementary and Integrative Medicine, University of Bern, 3012 Bern, Switzerland; 4 Society for Cancer Research, 4144 Arlesheim, Switzerland

**Keywords:** duckweed bioassay, in vitro bioassay, systematic negative control experiment, *Lemna gibba*, *praeparationes homoeopathicae*

## Abstract

European Pharmacopoeia monograph 2371 describes the production of homeopathic preparations. A specific efficacy of these preparations in high dilution levels is questionable in view of basic scientific principles. There is empirical evidence for such effects, for example in a Lemna-intoxication bioassay published 2010. To test the replicability and robustness of this bioassay, we conducted two experimental series (five independent blinded and randomised experiments each). The specimen of *Lemna gibba* L., clone-number 9352, were stressed in arsenic solution for 48 h (158 mg/L AsNa_2_HO_4_ (250 mg/L in series 2)), then grew in either As_2_O_3_ preparations produced according to Eu. Pharm. Monogr. 2371 or control solution. Comparing the area-related relative growth rate of day 3–9 (rgr 3–9) between treatment and control groups for each series showed differences that were not significant in series 1 (*p* = 0.10), significant in series 2 (*p* = 0.04) and significant in the pooled data of both series (*p* < 0.01). The effect direction (rgr 3–9 increase) was comparable to experiments of 2010, but the effect size was smaller, likely due to a changed light cycle. These results are not compatible with the hypothesis that the application of European Pharmacopoeia monograph 2371 results in pharmaceutical preparations without specific effects. Further studies are needed to investigate a potential mode of action explaining these effects.

## 1. Introduction

Preparations produced according to European Pharmacopoeia monograph 2371 and 1038 (*praeparationes homoeopathicae*, *ph*) [1] are being prescribed for therapeutic use by homeopathic and anthroposophic doctors and therapists, and are also publicly available in a large number of over-the-counter products. The use of *ph* is controversially discussed [2,3,4,5]. The view that *ph* induce specific effects beyond placebo is questioned, as it conflicts with current knowledge of pharmacology [6,7]. One of the main arguments is that homeopathic preparations do not contain a sufficient concentration of active substances to interact with cellular receptors, which in turn could cause specific effects in the human body [8]. Reports of successful treatments with homeopathic preparations are therefore often ascribed to non-specific effects, such as placebo effects [9].

There are biological test systems that employ either in vitro settings or plant experiments to test if homeopathic preparations might show effects that cannot be accounted for by placebo effects [10,11,12]. Jäger et al. [13] used a blinded and randomised test system, based on an ISO-certified bioassay [14], with arsenic-stressed duckweed *Lemna gibba* L. to investigate if potentised As_2_O_3_ (*Acidum arsenicosum* (HAB (German Homeopathic Pharmacopoeia)) also known as *Arsenicum album*) had an influence on the relative growth rate of plants after a phase of arsenic stress. They indeed observed significant differences between homeopathic treatment and control groups. Such unexpected results need to be replicated and critically assessed in terms of stability and reliability.

The aim of this publication is therefore to report on an internal replication trial of the experiments of Jäger et al. conducted in 2010 [13]. The experiments were performed by the same working group, but by a different experimenter in a different laboratory with slightly modified experimental parameters. We decided to change different parameters of the test system to test the robustness of the effects published by Jäger et al. [13]. This involved a change of light cycle for duckweed cultivation and experiments from 24 h continuous light to 16 h light and 8 h darkness. Additionally, the experiments took place in growth chambers that were specially designed for this bioassay. In these chambers, critical variables such as air movement as well as variation of light and temperature within the experimental set-up were controlled and monitored more strictly than in the chamber used for experiments in 2010. Furthermore, we tested two arsenic concentrations for stress induction: 158 mg/L AsNa_2_HO_4_ (as in the original trial) [13] and 250 mg/L AsNa_2_HO_4_ in a second series of experiments. To test the stability of the experimental set-up, we conducted two full series of systematic negative control (SNC) experiments [15].

## 2. Materials and Methods

### 2.1. Plant Cultivation

*Lemna gibba* L. clone no. 9352 cultures were cultivated in modified Steinberg medium as described in [14] (moSTM, all salts from Sigma Aldrich, Taufkirchen, Germany). The cultures derive from a laboratory culture at Aachen Technical University, Germany, 2001, and were further cultivated in our laboratory. Dr. Klaus Appenroth performed typisation of the duckweed clone in 2012. For long-time storage, axenic cultures were kept in 250 mL Erlenmeyer flasks (Schott, Mainz, Germany) with 100 mL modified Steinberg medium containing 1% agar (Sigma Aldrich, Taufkirchen, Germany) and 1% glucose (Sigma Aldrich, Taufkirchen, Germany). Axenic cultures were stored in a fridge in the dark at 4 °C and were renewed once a year.

Prior to an experiment, plants were given time to adapt to laboratory conditions. For this, two further cultivation forms were used. Duckweed from long-time storage was transferred to intermediate cultures. Intermediate cultures consisted of 500 mL Erlenmeyer flasks were filled with 250 mL of sterile modified Steinberg medium. They grew on acclimatised shelves (19 ± 1.5 °C air temperature in light and 16 ± 1.0 °C in dark (EBI 20 TH1 set, Ebro Electronic, Ingolstadt, Germany), 120 µmol photons s^−1^ m^−2^ PAR (Skye SKP200, light sensor SKP215, Skye Instruments Limited, Llandrindod Wells, UK), daylight lamps 412–715 nm (Philips T5 956 (chamber 1) or Philips T8 865 (chamber 2), Eindhoven, Netherlands), 55 ± 10% rel. humidity in light (EBI 20 TH1 set, Ebro Electronic, Ingolstadt, Germany)). Medium of intermediate cultures was renewed under sterile conditions every two weeks. Finally, plants were taken from intermediate culture and grown in 2 L modified Steinberg medium in 3.5 L glass vessels (Pyrex, Châteauroux, France) in acclimatised shelves (environmental conditions see above) to gain enough biomass for an experiment. The medium was renewed every week.

### 2.2. Preparation of Test Samples

On the day of an experiment, potentisation levels of As_2_O_3_ in the range of 6x–33x were produced by hand according to the multiple vessel method in pure water (distilled water (Multitron MT 40-1-E, BWT Holding GmbH, Mondsee, Austria) in Arlesheim; type 2 purified water (Millipore Elix Essential 10, Merck, Taufkirchen, Germany) in Freiburg). Between 5 and 10 a.m., potencies were produced by serial dilution and succussion of an *Arsenicum album* 5x dilution produced according to European Pharmacopoeia monograph 2371 (43% ethanol, corresponding to a concentration of 0.01 g/L As_2_O_3_, produced by Hevert, Nussbaum, Germany. The original series from 2010 [13] used an *Arsenicum album* 5x trituration from Weleda, Switzerland. For a discussion on a possible difference between dilution and trituration, see Section 4.3.3). At the company Hevert, the first produced potency level was 2x according to method 3.1.1 of European Pharmacopeia monograph 2371 and HAB monograph “*Acidum arsenicosum*”: one part *Arsenicum album* (quality according to European Pharmacopoeia monograph 1599) was dissolved under heat in ninety parts *Aqua purificata*, filtered and added to ten parts ethanol 86%. Succussion was performed by hand. The potency level 3x was produced using ethanol 15%. Potency levels 4x and 5x were produced with ethanol 43%. For the first potentisation level performed on the day of an experiment, 1.5 mL *Arsenicum album* 5x were diluted in 13.5 mL pure water in a 20 mL glass test tube (Duran glass, Schott, Mainz, Germany) with a glass stopper. The solution was shaken 12 times (within appr. 2 min). In the shaking process, the test tube was first moved up upside down, creating a laminar vortex by spinning the vessel; afterwards, the test tube was moved towards the floor at an amplitude of approximately 1.20 m to induce a chaotic fluid movement [13].

Different volumes were used for further potentisation levels. For the potentisation levels 7x–12x, 15 mL of the prior potency level were diluted in 135 mL pure water in a 250 mL Erlenmeyer flask (Duran glass, Schott, Mainz, Germany), respectively. Each potentisation level was accomplished in the same way (12 times vortex and chaotic movement). Further potentisation levels up to 33x were produced by diluting 35 mL of the prior level in 315 mL pure water in 500 mL Erlenmeyer flasks (Duran glass, Schott, Mainz, Germany) followed by the same succussion procedure. 

Water controls were prepared from the pure water of the same source as used for the potentisation level of As_2_O_3_ in 500 mL Erlenmeyer flasks (Duran glass, Schott, Mainz, Germany). Two kinds of water controls were used: unsuccussed water and succussed water (equivalent to water 1x). For the preparation of the succussed control, 350 mL of pure water was filled in a 500 mL Erlenmeyer flask (Duran glass, Schott, Mainz, Germany) and shaken exactly as described above.

Potencies were stored in opaque plastic boxes for a maximum of six hours until used in the experiment. For the experiments, the same potency levels as in the original series of 2010 (17x–18x, 21x–23x, 28x, 30x, and 33x) were used, with exception of 24x, which was omitted in the current experiments due to a smaller experimental field compared to the original experiments in 2010 (allowing for a total of 80 beakers compared to 100 beakers in the original experiments). Further information on the differences in the experimental arrangement is given in Section 2.4. Prior to an experiment, the flasks were coded (blinded) by a third person who was not further involved in the experiments. The code was only revealed after image analysis and the calculation of relative growth rates were accomplished.

For SNC experiments, all samples were prepared with pure unsuccussed water from the same water source as potentisation levels of As_2_O_3_. Samples for SNC experiments were not stored but directly transferred to beakers using a 500 mL Erlenmeyer flask (Duran glass, Schott, Mainz, Germany). 

### 2.3. Cleaning of Glass Vessels

Prior to the experiment, glass vessels were brushed under warm water, cleaned in a laboratory dishwasher without soap, autoclaved at 121 °C and air-dried. Between each step, they were rinsed with pure water (Type 1 purified water in Freiburg; deionised water (Enthärter Elite 2-RS, Septronline SL10, BWT Holding GmbH, Mondsee, Austria) in Arlesheim).

Erlenmeyer flasks that were used for potentisation were just brushed with pure water, rinsed with pure water and afterwards autoclaved and air-dried, to avoid remaining chemicals from the dishwasher.

### 2.4. Experimental Setting

**Figure 1:**
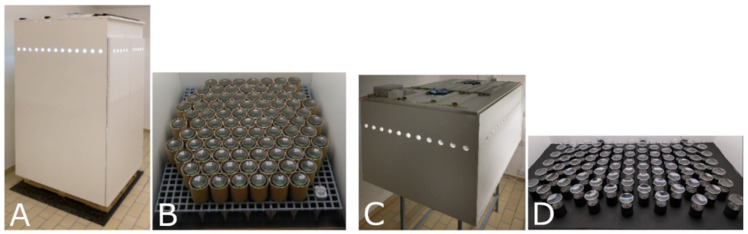
Growth chambers. (**A**) Chamber 1; (**B**) experimental field of chamber 1; (**C**) Chamber 2; (**D**) experimental field of chamber 2; due to different geometry of chamber 1 and chamber 2, the experimental field had to be modified to yield comparable conditions in temperature and air movement.

Experiments were conducted between 2016 and 2019, either in Freiburg (Germany) or in Arlesheim (Switzerland) (Table 1). Due to technical issues, a change in laboratory was necessary in 2018. Due to the same reason, two different growth chambers were used within the experimental series. Both chambers were specifically designed for the duckweed bioassay. They varied in geometrical shape and dimensions (Figure 1) but yielded comparable values for environmental variables (homogeneity of air flow, temperature, relative humidity and light). Two series of five main experiments and five SNC experiments were conducted. Table 1 shows the date, location and used growth chamber for all experiments. Growth conditions were similar in all chambers. The original and replication series differed in temperature and humidity due to the changed light regime: temperature of moSTM was at 21.5 ± 1.5 °C in light and 15.9 ± 1.1 °C in darkness in the replication series, and at 22.4 ± 0.3 °C in the original series. Humidity was at 32.5 ± 7.5% in light and at 52.5 ± 12.5% in darkness in the replication series, and at 68 ± 5% in the original series. Light intensity was set to 137 ± 11 µmol photons s^−1^ m^−2^ PAR, very similar to the original series [13]. Preliminary experiments had been performed to compare conditions in all used growth chambers.

For series 2 the concentration of arsenic as a stressor prior to experiments was increased from 158 mg/L to 250 mg/L AsNa_2_HO_4_. The hypothesis was that the effect size could be increased by a stronger stress induction prior to an experiment. The concentration of 250 mg/L was chosen as a medium stress, that was able to cause stronger reactions in the change of morphology of the plants but did not damage plants so severely that they would not survive.

The experimental procedure was comparable to the experiments of 2010 [13]: Prior to an experiment, healthy duckweed grew in a 2 L modified Steinberg medium (moStM). The last medium change was seven days prior to stress induction. Healthy duckweed (15 g fresh plant) that showed no lesion or chlorosis was transferred to a 2 L modified Steinberg medium containing an additional 158 mg/L (or 250 mg/L, respectively) AsNa_2_HO_4_. After 24 h, duckweed fronds with lesions, chlorosis and droplet-like morphology were discarded. After a further 24 h, duckweed plants without visible chlorosis or lesions were sorted according to their morphology into 3 groups of 85 plants each.

**Figure 2:**
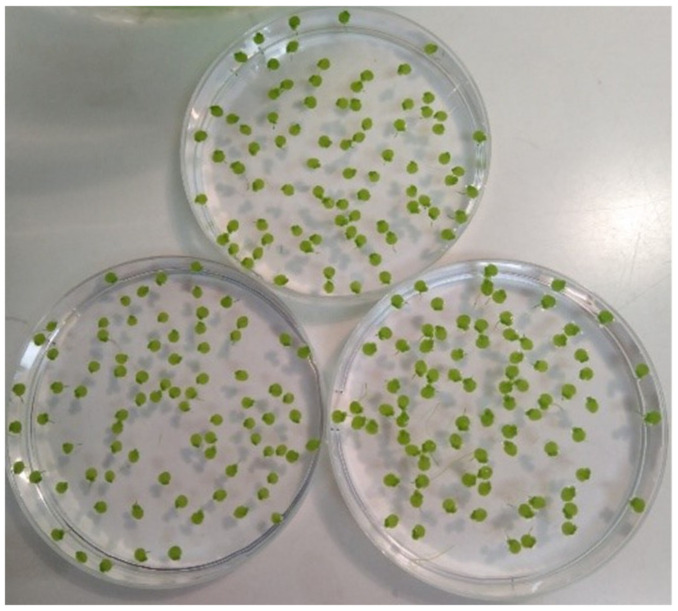
Sorting of duckweed prior to an experiment. Three groups of 85 fronds each were sorted. Duckweed fronds within each group had to be as similar as possible concerning symmetry and surface area. Additionally, fronds had to be non-chlorotic (whitening of the plant caused by the degradation of chlorophyll) and showing new small roots and daughter fronds. Figure 2 shows sorted plants stressed with 250 mg/L AsNa_2_HO_4_ for 48 h.

Within each of the three sorting groups, plants had to be as similar as possible in terms of their morphology, symmetry and surface area (Figure 2). One plant of each sorted group was put in a 150 mL beaker (Schott, Mainz, Germany) containing 50 mL double concentrated moStM and 50 mL of the potentised As_2_O_3_ or 50 mL water control. Volumes of 50 mL were transferred using a tilting automat (Duran glass, Schott, Mainz, Germany). Thus, each beaker contained three plants at the start of the experiment. An experiment comprised of 80 beakers, in 16 groups of 5 beakers each (with 3 experiments containing 14 groups, see Table 1). Eight groups were treated with homeopathic preparations of potentised As_2_O_3_ (17x, 18x, 21x, 22x, 23x, 28x, 30x, and 33x). Four groups contained unsuccussed water controls and four groups contained succussed water controls (in three experiments, there were three succussed and three unsuccussed water control groups, see Table 1). 

Additionally, up to five beakers were used as open-label controls in each experiment (see Table 1). These open-label controls contained just moStM and one duckweed plant with three fronds (leaf) that was never exposed to AsNa_2_HO_4_. The plant was chosen according to its surface area that was supposed to be comparable to the surface area of the three stressed fronds used in the other beakers. By that, the starting surface area should be nearly the same for all beakers and the variance in growth rates was further reduced, resulting in more comparable controls. These additional open-label beakers were used as a measure for growth reduction induced by arsenic stress and control for the health state of duckweed prior to arsenic treatment. They were not included in statistical analyses. 

Stress induction was defined in terms of morphological changes in the plants after exposure to arsenic. The sorting of plants was based on those morphological changes. The damage rate caused by the stress was just an additional measure. The formula used to calculate the damage rate was changed compared to Jäger et al., 2010 [13]. In that publication, arsenic stress was calculated by comparing relative growth rates of plants that were stressed by AsNa_2_HO_4_ for the whole duration of the experiment and of plants that had no contact with arsenic at all. In the present publication, we compared relative growth rates (rgr) of the AsNa_2_HO_4_-pre-treated water control plants (rgr_AsH2O_) with relative growth rates of plants that never had any contact with AsNa_2_HO_4_ (rgr_NoAs_). The formula for growth reduction is:

r [%] = rgr_NoAs_/rgr_AsH2O_ × 100. (1)

We also recalculated the stress rate data of Jäger et al. 2010 [13] with the current formula to adequately compare toxicity levels in the original and in the current study. Reduction in growth rate within an experimental series was calculated from numerically pooling data for water controls and non-stressed duckweed for all experiments (five verum and five SNC experiments of each series). 

Each beaker was covered by a watch glass to reduce evaporation. Additionally, each beaker was wrapped into a black paper ring up to the water surface in the beaker to reduce the influence of scattered light. 

To ensure that differences in relative growth rate between groups were not due to differences of environmental variables in the chambers, a separate randomisation scheme was developed for each chamber to reduce variability and to exclude systematic errors due to unavoidable gradients of environmental factors within each chamber (light, temperature, air movement, etc.). Randomisation schemes were developed based on and tested in preceding SNC experiments.

Each beaker was photographed with a camera (Canon EOS 1200D, Photo-objective Canon EF-S 60/2.8, Canon, Tokyo, Japan) while standing on an LED-light source (NW-Pad22 112 LED, 12W, 5600/3200K dimmable LED-panel, Neewer, Guangdong, China on experimental days 0, 3 and 9. The surface area of the plants was measured in the images using ImageJ macros [16]. For series 1, the ImageJ plugin “Immunohistochemistry (IHC) Image Analysis Toolbox” [17] was implemented as well to detect the green values of duckweed fronds. For series 2, the analysis was based on the wand tool instead of the “IHC Image Analysis Toolbox” due to later developed chlorosis (whitening of the plant caused by the degradation of chlorophyll) in a few plants. 

Based on the surface area, the relative growth rate for each beaker was calculated for two different time periods (0–3 days and 3–9 days) according to the formula

rgr = ((ln(x_t2_)) − (ln(x_t1_))/(t_2_ − t_1_)) (2)

where rgr = relative growth rate, x_t1_/x_t2_ = surface area of plants at time point one/two, t_1_/t_2_ = time point one or two in days.

### 2.5. Systematic Negative Control (SNC) Experiments

SNC experiments were used to investigate the stability of the test system. Instead of treatment groups, an SNC experiment uses unsuccussed water controls in each group. Any differences between groups in SNC experiments are due to the biological variability of plants and differences in environmental factors within the experimental set-up. If the same statistical analysis is used for the SNC as for a “verum” experiment, a non-significant result corresponds to a stable test system. Every SNC was allocated to a verum experiment. For the allocation process, two groups of five beakers were excluded from the analysis in two SNC of the first replication series and three SNC of the second replication series to match the number and position of beakers in the according verum experiments (Changes are shown in Table 1). 

### 2.6. Missing Data

Due to careful experimental management, there are no missing values in the two experimental series and the corresponding SNC experiments.

### 2.7. Statistical Analysis and Software

Statistical analysis was performed by A.Ü. with the software JMP 14 (SAS Institute Inc., Cary, NC, USA). Relative growth rates were calculated in Excel 2010 (Microsoft, Redmond, WA, USA). Image analysis was performed in ImageJ (Macros see Supplement A). A second person (S.B.) recalculated the statistical evaluation of all the main experiments (but not coefficients of variance) using Statistica 13.3 (Tibco Software Inc., Palo Alto, CA, USA).

For statistical analysis, homoscedasticity was calculated using the Levene test. A two-way ANOVA was calculated for the treatment group and experimental day. The significance level was set at 5%. For analysis of numerically pooled series, after testing for homoscedasticity with Levene test, a three-way ANOVA was calculated for the treatment group, experimental day and experimental series. A Fisher LSD post hoc test was calculated for the interaction of treatment group and experimental series using least square means.

SNC experiments were statistically analysed in the same way as verum experiments by forming pseudo-treatment and pseudo-control groups (for alignment of SNC experiments and verum experiments see Table 1).

## 3. Results

### 3.1. Measure for Arsenic Stress, Variance Coefficient

To determine the effect of arsenic pre-treatment, the relative growth rates of the open-label duckweed control (rgr_NoAs_), which was never exposed to arsenic, were compared to the growth rates of *Lemna gibba* L. which were pre-treated with arsenic, but not treated with potentised As_2_O_3_ (rgr_AsH2O_), for the late time period (3–9 days).

In the first series (using 158 mg/L AsNa_2_HO_4_) reduction of relative growth rate was 2% (rgr_NoAs_ = 0.316 and rgr_AsH2O_ = 0.310). In the second series (with 250 mg/L AsNa_2_HO_4_), growth reduction was 10% (rgr_NoAs_ = 0.345; rgr_AsH2O_ = 0.309). In the original series from 2010 [13], growth reduction by arsenic stress was 1%: rgr_NoAs_ = 0.420 and rgr_AsH2O_ = 0.416 (recalculated data).

Table 2 shows the coefficients of variance (CV) of all SNC experiments in both series for early (day 0–3) and late time periods (day 3–9). In order to determine the degree of variability between randomised groups, CV was calculated as the standard deviation of randomisation group means/mean of randomisation group means.

**Figure 3:**
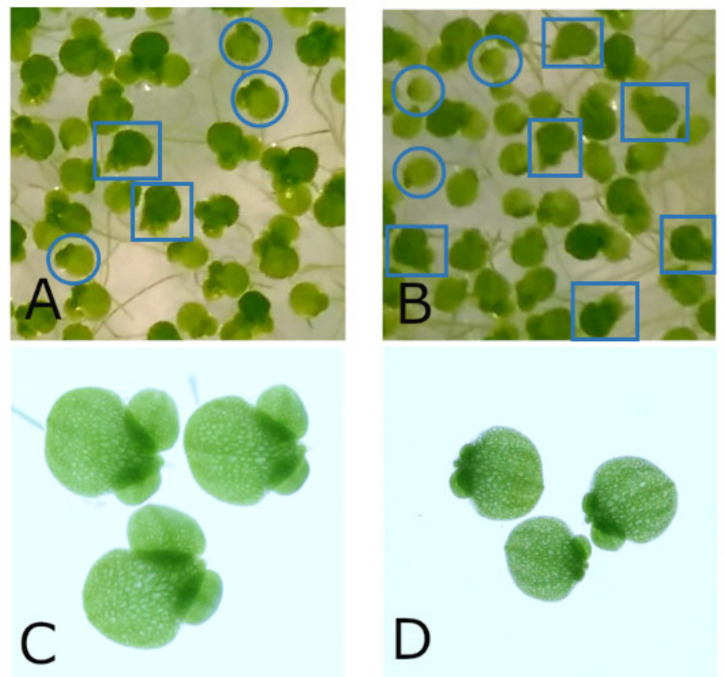
Morphological changes induced by different concentrations of AsNa_2_HO_4_ on *Lemna gibba* L. by 48 h incubation resulted in the separation of mother and daughter fronds (leaf-like structure). (**A**) 158 mg/L AsNa_2_HO_4_ plants prior to sorting (**B**) 250 mg/L AsNa_2_HO_4_ plants prior to sorting (**C**) 158 mg/L AsNa_2_HO_4_ sorted and (**D**) 250 mg/L AsNa_2_HO_4_ sorted. (**C**,**D**) have the same scale. Blue circles indicate plants considered optimal for experiments. Diameter of fronds and daughter fronds are smaller after stress with 250 mg/L AsNa_2_HO_4_ (see **A**) compared to 158 mg/L AsNa_2_HO_4_ (see **B**). Blue rectangles indicate severely stressed plants, recognisable by their droplet-like morphology. Their number increases with a higher arsenic stress. More severely stressed plants have a tendency to develop chlorosis (whitening due to loss of chlorophyll).

The growth reduction caused by arsenic stress prior to an experiment differed between the two series. In series 1 the arsenic stress of 158 mg/L AsNa_2_HO_4_ caused a reduction of relative growth rates of less than 2%, but a clearly visible change in morphology of plants (Figure 3) as observed elsewhere [13]. Mother- and daughter-fronds (frond = leaf-like structure) tended to separate from each other and lose their roots. Some plants developed chlorosis. After 48 h, small daughter fronds of the second generation and very short roots were visible on the former daughter fronds. In series 2 (250 mg/L AsNa_2_HO_4_), plants reduced their relative growth rate by 10% compared to non-stressed plants. Similar changes in morphology as in series 1 were visible, but daughter fronds of the second generation and newly grown roots were smaller after 48 h and the rate of fronds showing signs of chlorosis increased (see Figure 3B).

### 3.2. Water Controls C0, C1

Two kinds of water controls (unsuccussed vs. succussed) were used to investigate whether the process of succussion of water itself had an influence on the relative growth rate of duckweed. In each experimental series, we therefore compared unsuccussed and succussed water controls, numerically pooling data of the five verum experiments of each series. 

There was no significant difference between the two water control groups in both experimental series for both time periods (*p* = 0.646 (day 0–3) and *p* = 0.776 (day 3–9) in series 1; *p* = 0.800 (day 0–3) and *p* = 0.437 (day 3–9) in series 2). Therefore, as had been defined a priori, both water control groups were numerically pooled in further analyses to increase statistical power.

### 3.3. Systematic Negative Control (SNC) Experiments

In SNC experiments, all treatment groups consisted of unsuccussed water. At the same time, the same experimental conditions, randomisation codes and statistical analysis as in the respective verum experiment were applied. This allows investigating the stability of the test setting. Since plants undergo seasonal changes that could influence the stability of the test system, an SNC experiment was performed for every verum experiment. Furthermore, a comparable statistical power for verum and SNC experiments was achieved. 

**Figure 4:**
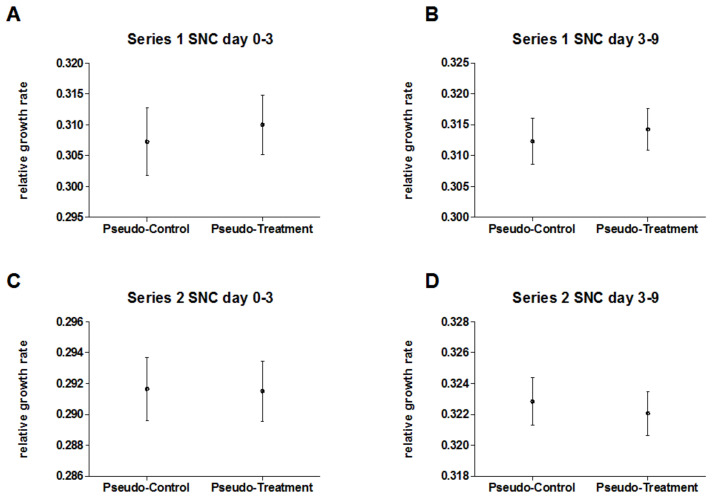
Mean relative growth rates (±95% CI) of duckweed of five SNC experiments in each series (1/2) for numerically pooled data of pseudo-treatment (n = 200) and pseudo-control group (n = 170) for (**A**) series 1 (early time period, day 0–3); (**B**) series 1 (late time period, day 3–9); (**C**) series 2 (early time period, day 0–3); and (**D**) series 2 (late time period, day 3–9).

Figure 4 displays relative growth rates of pseudo-treatment groups and pseudo-control groups for the SNC experiments of both experimental series at early and late time periods. There was no significant difference between the (identically treated) groups for any time period and/or experimental series (Supplement B), indicating a stable test system.

### 3.4. Experimental Series 1

**Figure 5:**
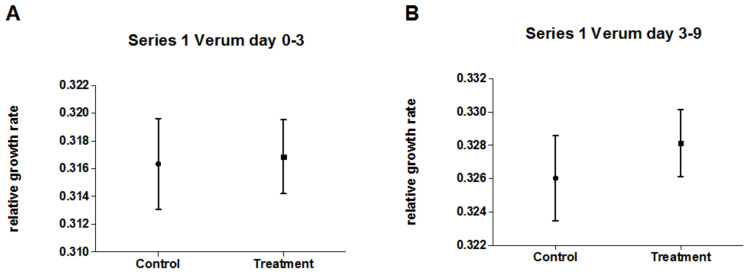
Mean relative growth rate (±95% CI) of duckweed treated with either potentised As_2_O_3_ (potency levels 17x–18x, 21x–23x, 28x, 30x, 33x, numerically pooled, n = 200) or water as control (unsuccussed and succussed water samples, numerically pooled, n = 170) in the 5 independent experiments of series 1. (**A**) Early time period (day 0–3), (**B**) Late time period (day 3–9).

**Figure 6:**
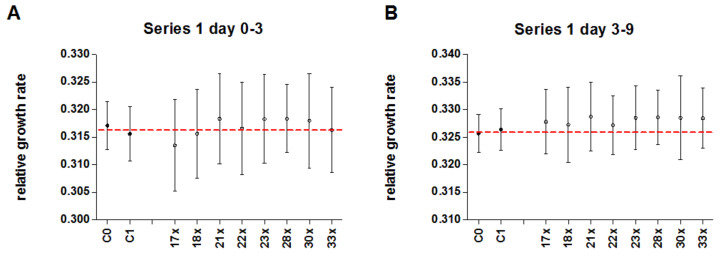
Mean relative growth rate (±95% CI) of duckweed treated with either potentised As_2_O_3_ (potency levels 17x–18x, 21x–23x, 28x, 30x, 33x, numerically pooled, n = 25 each) or water as control (unsuccussed and succussed water samples, numerically pooled, n = 85 each) in the 5 independent experiments of series 1. (**A**) Early time period (day 0–3), (**B**) Late time period (day 3–9). The red line represents the mean of water controls.

In series 1, duckweed was pre-treated with 158 mg/L AsNa_2_HO_4_ as stressor, and afterwards treated with either potentised As_2_O_3_ (17x–18x, 21x–23x, 28x, 30x, and 33x) or unsuccussed or succussed water as controls. Figure 5 shows relative growth rates of duckweed for the early (day 0–3; *p* = 0.97) and late time period (day 3–9; *p* = 0.10) for numerically pooled treatment and control groups. In Figure 6, means for single treatment groups (succussed and unsuccussed water controls and single potency levels), averaged over five independent experiments are displayed. There were no significant differences between pooled or single treatment groups and controls (Table 3 and Table 4). There is a slight trend for higher relative growth rates in pooled treatment groups of the late time period (+0.64% compared to controls). There were strong differences between single experiments and no significant interaction between treatment and experiment number.

### 3.5. Experimental Series 2

**Figure 7:**
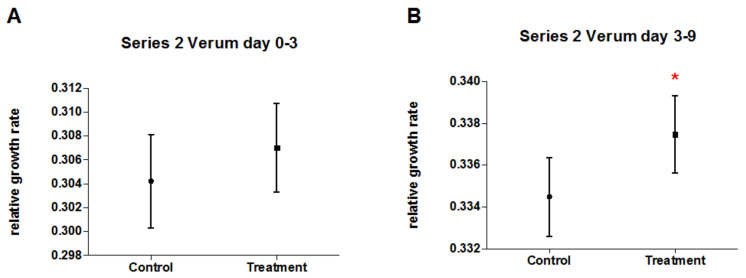
Mean relative growth rate (±95% CI) of duckweed treated with either potentised As_2_O_3_ (potency levels 17x–18x, 21x–23x, 28x, 30x, 33x, numerically pooled, n = 200) or water as control (unsuccussed and succussed water samples, numerically pooled, n = 170) in the 5 independent experiments of series 2. (**A**) Early time period (day 0–3), (**B**) Late time period (day 3–9), * *p* < 0.05.

**Figure 8:**
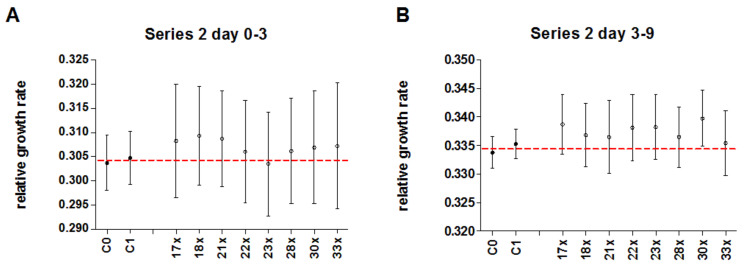
Mean relative growth rate (± 95% CI) of duckweed treated with either potentised As_2_O_3_ (potency levels 17x–18x, 21x–23x, 28x, 30x, 33x, n = 25 each) or water as control (unsuccussed and succussed water samples, n = 85 each) in n = 5 independent experiments of series 2. (**A**) Early time period (day 0–3), (**B**) Late time period (day 3–9). The red line represents the mean of water controls.

In series 2, duckweed was pre-treated with 250 mg/L AsNa_2_HO_4_ as stressor, and afterwards treated with either potentised As_2_O_3_ (17x–18x, 21x–23x, 28x, 30x, and 33x) or unsuccussed or succussed water as controls. After numerical pooling of treatment and control groups, there was no significant difference in the early time period (day 0–3; *p* = 0.8, Table 5, Figure 7A) and a significantly higher relative growth rate (+0.89%) of duckweed treated with potentised As_2_O_3_ compared to the control groups in the late time period (day 3–9; *p* = 0.04, Table 5, Figure 7B). There were no significant differences between single treatment groups (Table 6, Figure 8). In all analyses, we observed a strong influence of experimental day and a non-significant interaction between experiment number and treatment group.

### 3.6. Pooled Data of Experimental Series 1 and 2

**Figure 9:**
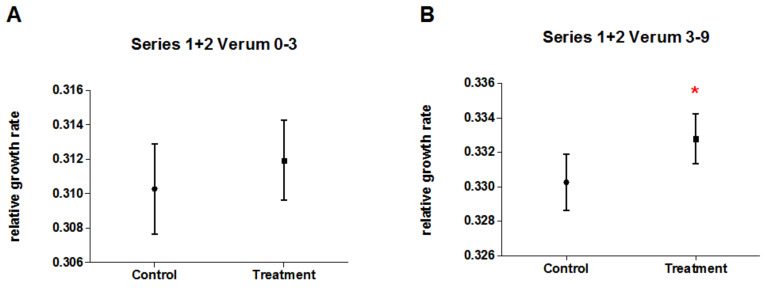
Mean relative growth rate (±95% CI) of duckweed treated with either potentised As_2_O_3_ (potency levels 17x–18x, 21x–23x, 28x, 30x, 33x, numerically pooled, n = 400) or water as control (unsuccussed and succussed water samples, numerically pooled, n = 340) in the 10 independent experiments of series 1 and series 2. (**A**) Early time period (day 0–3), (**B**) Late time period (day 3–9), * *p* < 0.05.

**Figure 10:**
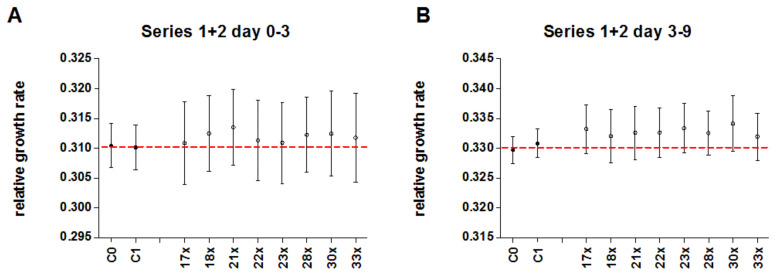
Mean relative growth rate (±95% CI) of duckweed treated with either potentised As_2_O_3_ (potency levels 17x–18x, 21x–23x, 28x, 30x, 33x, n = 50 each) or water as control (unsuccussed and succussed water samples, n = 170 each) in the 10 independent experiments of series 1 and 2. (**A**) Early time period (day 0–3), (**B**) Late time period (day 3–9). The red line represents the mean of water controls.

In addition to the statistical analysis of both single series, numerically pooled data of both series were analysed as well. With the increase in data points by numerical pooling of both series, a higher statistical power was aimed for. After numerical pooling of treatment groups, there was no significant difference in the early time period (day 0–3; *p* = 0.85, Table 7, Figure 9A) and a significantly higher relative growth rate of duckweed treated with potentised As_2_O_3_ compared to the control groups in the late time period (day 3–9; +0.77%, *p* < 0.01, Table 7, Figure 9B). There were no significant differences between single treatment groups (Table 8, Figure 10). In all analyses, we observed a strong influence of the experimental day and no significant interactions between the treatment group and other parameters.

## 4. Discussion

In the present experiments, duckweed cultures were pre-treated with 158 or 250 mg/L AsNa_2_HO_4_ as stressor, and afterwards treated with either potentised As_2_O_3_ (potency levels 17x–18x, 21x–23x, 28x, 30x, and 33x) or control solutions (unsuccussed and succussed water samples). Two series—differing regarding arsenic stress induction—with five independent experiments and five independent systematic negative control (SNC) experiments each were carried out. The SNC experiments confirmed the stability of the test system. For the early growth period (day 0–3) no effects of the treatment with potentised As_2_O_3_ were observed in any series. In the late growth period of the test (day 3–9), significant growth-increasing effects through potentised As_2_O_3_ were observed in the second series and a similar trend in the first series. Numerically pooled data of the late time period (day 3–9) of both series yielded statistical evidence (*p* < 0.01) for specific effects of potentised As_2_O_3_ on the relative growth rate of duckweed.

### 4.1. Influence of Arsenic Stress Level in Series 1 and 2

The main difference between series 1 and series 2 was the arsenic concentration used prior to an experiment. In series 1, we used the same concentration of 158 mg/L AsNa_2_HO_4_ as in the original series of Jäger et al. in 2010 [13], but changed the light cycle and growth chambers. In series 2, we used 250 mg/L AsNa_2_HO_4_ prior to an experiment to induce greater stress in the plant, causing more severe changes in their morphology (see Section 3.1).

#### 4.1.1. Morphology of Plants and Stress Level

The objective of stress induction prior to an experiment was to have plants in a state that might be similar to an illness. Potentised preparations are argued to have an influence on restoring an equilibrium state that is defined as being healthy. The stress might disrupt this equilibrium, leading to a stronger response to the treatment. 

For replicability reasons, a stable stressor was chosen, which introduced similar changes in the morphology and growth rate of the plants for experiments at different time points independent of seasonal changes in the plants. The stressor AsNa_2_HO_4_ was chosen after screening different stressors prior to the experiments of 2010 [13]. Additionally, the induced stress level had to be high enough to severely influence the plants, but not too high, since they still should be able to recover after the stress induction. For those reasons, a stress level of 158 mg/L AsNa_2_HO_4_ under continuous light was used in the experiments of 2010 [13]. As shown in Section 3.1, plants reacted to arsenic stress by discarding their roots and separating daughter fronds. The growth rate of plants was inhibited during arsenic exposure. The comparably small growth reduction of experiments (1–2%, see Section 2.4 and Section 3.1) is due to the fact that *Lemna gibba* L. are fast-growing, robust pioneer plants [18]. Since the reduction of growth rate in experiments was measured after removing the source of stress, plants were already again able to compensate by an increased growth rate. 

Nonetheless, biochemical changes in the plants were likely to be still active as arsenic species introduce multiple stress reactions in plants that change their biochemistry [19]. *Lemna gibba* L. is able to hyperaccumulate arsenic, but above a certain concentration, damages are visible. In general, several influences on biochemical pathways by arsenic in duckweed are known and could be used as a starting point for in-depth molecular analyses of the plants’ stress level: First, arsenic(V) compounds act in competition to phosphate molecules (Pi), disrupting energy acquisition [20,21]. As part of the detoxification process, arsenic(V) is reduced to arsenic(III) generating ROS (reactive oxygen species). The higher oxidative stress damages cell walls and increases protein degradation [22]. Additionally, carbon sources (partly from the degradation of chlorophyll, leading to chlorosis) are used to increase the production of different antioxidants, such as phytochelatines, glutathione or ascorbic acid [23]. As a third factor, arsenic(III) binds with a high affinity to sulphur, inactivating enzymes [24]. All these factors together cause a change in morphology in plants. 

Not all the plants’ biochemical changes are necessarily correlating with the intensity of changes in their morphology. This might be a reason why under a changed light cycle compared to experiments of 2010 (16:8 h light compared to 24:0 h light in 2010) [13], the effect size of treatment with potentised As_2_O_3_ was lower, even though stressed plants were morphologically comparable. Observing levels of antioxidants, ROS caused damages or uptake of phosphate might be interesting additional factors for characterising the stress for plants more closely. 

#### 4.1.2. Stress Level and Effect Size

In the two present experimental series, the effect size of treatment with potentised As_2_O_3_ compared to water controls seemed to increase with increasing stress level (+0.64% for 158 mg/L AsNa_2_HO_4_ and +0.89% for 250 mg/L AsNa_2_HO_4_ pre-treatment). Nonetheless, the effect size in both series was lower compared to the original series of 2010 (+1.21% in [13]). A possible hypothesis to explain this difference is that stress level as well as light cycle, play a decisive role in the biological response to potentised preparations. 

Hribar-Marko et al. [25] published a series of experiments in which wheat seeds (*Triticum aestivum*) were pre-treated with three different concentrations (10^−3^, 10^−4^, and 10^−5^ parts per weight) of gibberellic acid, a growth promotion factor in plants. The pre-treatment caused an increase in stalk length after seven days. When seeds were grown in potentised gibberellic acid 30x (or water 30x as control) after pre-treatment, stalk length tended to decrease. The effect in decreased stalk length by homeopathic preparations was significant in a control without pre-treatment. A medium pre-treatment of 10^−4^ parts per weight gibberellic acid showed the strongest decrease in stalk length within experiments with pre-treatment, but it was less pronounced than in experiments without pre-treatment. There was no difference detectable for pre-treatment with 10^−3^ parts per weight. In these experiments, pre-treatment had an influence on the effect size of potentised substances, but there was no clear linear correlation for the concentration of pre-treatment. 

In 2019 and 2021, Jäger et al. [26,27] published experiments with duckweed (*Lemna gibba* L.) that were either pre-treated for 48 h with a high concentration of mercury chloride (5 mg/L), causing severe changes in plant morphology and a reduction of area-related relative growth rate, or that were pre-treated with a lower concentration of mercury chloride (2.5 mg/L), causing less severe damage. Afterwards, plants were treated with different potency levels of *Mercurius corrosivus* (24x–30x, compared to unsuccussed water and water 1x). A pre-treatment with 5 mg/L HgCl_2_ led to a significant growth rate reduction in groups treated with *Mercurius corrosivus* potencies in the early time period (day 0–3). Whereas pre-treatment with 2.5 mg/L HgCl_2_ led to a significant increase in growth rate in groups treated with potentised *Mercurius corrosivus* in a late time period (day 3–9). Jäger et al. proposed a hypothesis of a non-linear correlation between stress level and effect size. With an increase in stress level, the effect size would follow a sinusoid curve. Thus, a very low-stress level would correlate with a very low effect size. Increasing stress levels would induce higher effect sizes, and even higher stress levels would lead to a turning point and to an inversion in effect direction.

An application of this hypothesis to the present experiments would predict that a stress level between 158 and 250 mg/L AsNa_2_HO_4_ would lead to a higher effect size. An even higher stress level would be expected to cause an inversion of effect direction. Further experiments with the present test system of arsenic-stressed duckweed could be designed to test this hypothesis.

#### 4.1.3. Variability and Stress Induction

The coefficient of variance (CV) was slightly lower in series 2 compared to series 1 (see Section 3.1), though a higher CV was expected for series 2 due to the higher arsenic stress applied. We hypothesise that more stringent handling and sorting of plants prior to an experiment (due to increased experience of the experimenter) led to a reduced CV. Accordingly, series 2, showed more pronounced differences between treatment and control groups in a statistical sense. 

The CV in the original series [13] (n = 5 experiments, late time period, day 2–6, CV = 1.74%) was higher than in series 1 (n = 5 experiments, late time period, day 3–9, CV = 1.14%) and series 2 (n = 5 experiments, late time period, day 3–9, CV = 1.10%). The lower CV in series 1 and series 2 might be caused by stricter control of surrounding conditions in the new, specially constructed growth chambers that were introduced for both series. 

In the original series [13], the effect of potentised As_2_O_3_ was larger compared to the present replication series 1 and 2. The smaller coefficient of variance compared to the original series did not correlate with higher effect sizes. Thus, other factors were more relevant when comparing original and replication series (see Section 4.3).

### 4.2. Stability of the Experimental Set-Up

SNC experiments of the two series were evaluated in the same way as the experiments with potentised As_2_O_3_. There were no significant differences detected in any series of SNC experiments for neither early nor late time periods. These results are in favour of a stable test system and of a valid statistical approach, and exclude to a very high degree that the significant differences in the verum experiments should be considered as false-positive results.

We observed no differences between succussed and unsuccussed water in their effect on the relative growth rate of duckweed. The ratio for using two kinds of water controls is that succussion (shaking) could change physico-chemical characteristics of water, e.g. by an increased amount of dissolved oxygen and carbon dioxide in the water, and a corresponding slight change in pH. Additionally, higher amounts of dissolved sodium, silica and other compounds from the glass walls of the potentisation vessel were measured after succussion [28]. With the additional succussed water control, changes in the relative growth rate of plants due to these possible physico-chemical changes are accounted for. In our case, the succussion procedure in itself did not influence the test system.

We took multiple measures to reduce the influence of possible confounders and occurrence of false-positive results: the sample size for controls was set to be nearly equal to treatment groups, ensuring statistical comparability of groups. Surrounding conditions (temperature, light intensity, air movement, air humidity) of experiments were monitored and controlled to ensure comparability between experiments. Additionally, all experiments were conducted randomised and blinded to exclude the unconscious influences of the experimenter. The stability of the test system and the statistical procedures were monitored by SNC experiments and strict documentation of experimental procedures, including documentation of the image analysis process. Two researchers independently performed the statistical evaluation with different statistical software (A.Ü. with JMP 14 and S.B. with Statistica 13.3). Therefore, we conclude that the significant difference between treatment and control groups is most likely not a false-positive result, but a reaction to the treatment.

### 4.3. Comparison of Series 1 and 2 to the Original Experiments of 2010

#### 4.3.1. Cultivation Conditions and Growth Chambers

The differences in cultivation conditions between series 1 and series 2 were small, as documented by SNC experiments and observing surrounding conditions (see Table 1). The experimental settings were also comparable to the original series of 2010 concerning duckweed clone, light spectra, temperature and plant cultivation media [13]. 

We decided to change the light cycle from continuous light in the original series to 16 h light and 8 h darkness. The shorter light cycle had no visible influence on the stress reactions of plants to arsenic stress with 158 mg/L AsNa_2_HO_4_. There was still a comparable change in morphology and no relevant reduction of growth rate, as in the original series (original series (158 mg/L AsNa_2_HO_4_): 1% reduction of relative growth rate; series 1 (158 mg/L AsNa_2_HO_4_): 2% reduction of relative growth rate (see Section 3.1)). We had hypothesised that a light rhythm mimicking natural conditions more closely would lead to a stronger reaction to potentised preparations. Additionally, a change of light and dark periods induces synchronisation of circadian rhythms in duckweed [29]. Moreso, here we had hypothesised that plants might react stronger to homeopathic treatments when their circadian rhythm is synchronised between their single cells. We must, however, conclude that these hypotheses were most probably wrong since the effect size in the present replication series was smaller than in the original series (+0.77% compared to +1.21%). Even if differences in stress reaction according to changes in morphology and growth rate were not detectable between the two light cycles, biochemical differences might have occurred and influenced the effect size (see Section 4.1.1).

#### 4.3.2. Image Analysis Software

Since the image analysis software (Scanalyzer, duckweed analytic software, version 4, LemnaTec, Aachen, Germany) that was used in 2010 [13] was no longer available in our group, we decided to use open-source alternatives for image analysis. For the first series, an ImageJ macro [16] was employed, that used a green value for object identification. As shown in Supplement A, we used an ImageJ plugin of (“Immunohistochemistry (IHC) Image Analysis Toolbox”) [17] to pick green values of plants from the photos and used the segmentation function of this plugin for the detection of plant surface area. Segmentation was corrected by hand, if necessary, and masks of the final segmentation were stored. A comparison between masks and original photos enables an estimation of the precision of segmentation.

For series 2, this macro was not accurate enough because of single chlorosis formation (a whitening of the plant due to a loss of chlorophyll) on single fronds, especially at the border of the fronds. The chlorotic parts of the plant were not detectable by a green value analysis. Correction of the frond borders by hand was assumed error-prone. Therefore another ImageJ macro based on the use of the wand tool for frond detection was used instead. Both macros were compared on the same set of images. The difference between surface area detection of both macros was negligible. Therefore, we assumed comparability of surface area detection with both approaches. Masks of segmentation were also stored by this second macro. Therefore, the accuracy of segmentation is evaluable as well.

#### 4.3.3. Further Possible Influences on Effect Size

Compared to the original series [13], the results showed the same effect direction, but a smaller effect size. In all series, no influence of potentised As_2_O_3_ was observed in the first growth period; in the second growth period, potentised As_2_O_3_ led to an increase in relative growth rate. This increase was on the average 1.21% (*p* < 0.001) in the original series, 0.64% (*p* = 0.097) in series 1 and 0.89% (*p* = 0.044) in series 2, respectively. 

Due to technical reasons, there were some changes necessary in used materials and in the experimental setting compared to the original series. In the original series, *Arsenicum album* potentisation levels were produced from a 5x *Arsenicum album* trituration (Weleda, Arlesheim, Switzerland). Because this trituration was no longer available, 5x *Arsenicum album* dilutions (43% ethanol, Hevert, Nussbaum, Germany) were used instead. Both manufacturers produce their homeopathic preparations according to the European Pharmacopeia monograph 2371 [1]. From that point of view, the preparations should be comparable. However, there is the possibility that efficacy depends on details in the manufacturing process (trituration or dilution). We are not aware of any publication reporting on investigations of possible differences in the efficacy of trituration or dilution of potentised substances in biological or in vitro assays. 

There were also changes in the location of laboratories and in the experimenter, compared to the original study. Both factors are discussed as potential modulating factors on the experimental outcome. Therefore, they are advised to be reported in reporting guidelines for homeopathic basic research [30]. It is possible that other variables are correlated to the factors of experimenter and location. 

Some publications on plant-based test systems investigating the effects of potentised substances reported on possible modulating factors on the experimental outcome. The following section gives an overview of the literature and draws conclusions for the arsenic-stressed duckweed bioassay.

In a series of experiments, Hamman et al. [31] tested different harvesting lots of barley seeds (*Hordeum vulgare* L.) in an in vitro germination bioassay using potentised gibberellic acid and detected different effects depending on the seed lot. Therefore, they hypothesized that vigour (the overall ability of seeds to germinate and grow) of the harvesting lot might modulate the response to potentised preparations. 

An Italian research group reported on trials with an arsenic-stressed wheat seed (*Triticum durum* L.) germination model using potencies of As_2_O_3_. They found a significant increase in the germination rate and stalk length after seven days for treatment with As_2_O_3_ 45x compared to water control groups [32,33]. Two publications reported on external replication trials with this test system in Switzerland. They observed a decrease in stalk length after seven days of germination for treatment with As_2_O_3_ 45x compared to controls. They were not able to identify the reasons for this effect inversion. Testing for the influence of different factors, such as seed varieties, geographic location and seed sensitivity to arsenic poisoning showed no correlation to effect size or direction [34,35]. 

In a test system with wheat seeds (*Triticum aestivum*, Capo variety), potentised gibberellic acid was used in 16 experiments, performed by 9 experimenters. Seasonality was correlated to effect size [36]. Inhibiting effects on stalk length after seven days compared to water controls were reliably detectable in autumn but neither in winter nor spring. Scherer-Pongratz et al. [37] reanalysed 30 experiments performed by 6 experimenters with a similar test system using wheat seeds (*Triticum aestivum*) and potentised silver nitrate. They hypothesised that effects (enhanced growth of stalk after seven days compared to control groups) were more pronounced in experiments with a medium stalk length in control groups. 

All these modulating factors were previously detected in germination models. However, the duckweed test system uses continuously asexually spreading adult plants. Therefore, the harvesting lot cannot be a modulating factor. It is, however, of interest to analyse possible seasonal influences. Correlation calculations within all three series of arsenic-stressed duckweed revealed no significant interactions of seasonality and effect size, however (data not shown).

Majewsky et al. [38,39] identified a specific morphology as a relevant modulating factor in a non-stressed duckweed-bioassay (*Lemna gibba* L.). Only when plants were in the state of gibbosity (enlarged aerenchym) did treatment with potentised substances (potentised gibberellic acid or silver nitrate) show significant influence on the growth rate of duckweed compared to water controls after seven days. Plants with a flat aerenchym did not react to treatment. There is no clear correlation between gibbosity and seasonality; however gibbous plants are more common in autumn than in summer under natural conditions [40]. In our experiments, plants were discarded if they showed signs of gibbosity to ensure smaller variability between experiments.

Hypothetically, mutations of the plants over time could be a source for changes in reactivity to stress and to potentised preparations. The duckweed strain we used (*Lemna gibba* L. clone-number 9352) was cultivated for more than ten years in our laboratories between the original series [13] and the present two new series. Genetic investigations revealed that duckweed has a very low mutation rate [41,42]. In our experiments, induction of flowering was inhibited by experimental conditions. Additionally, plants reacted with a similar morphological change as they did in the original series. Nonetheless, a comparison of the genetic typisation that was done in 2012 could be repeated to investigate this question further.

### 4.4. Outlook

Based on the available data, we see a considerable potential of the present arsenic-stressed duckweed bioassay to be developed into a standardised test system, which could be used to assess the specific efficacy of preparations produced according to European Pharmacopoeia monograph 2371 (*preparationes homoepathicae*). To achieve this, the bioassay has to be further optimised. Therefore, future experiments should compare the influence of continuous light to a 16:8h light cycle. By working in parallel, other confounding factors, such as the age of plant cultures, slight changes in medium, surrounding conditions or seasonal changes can be excluded. We also suggest testing the hypothesis of a sigmoid correlation of stress level to effect size as described in Section 4.1.2 and in Jäger et al. [26,27]. After optimization, preparations from different manufacturers could be investigated, and also further research questions of pharmaceutical interest (e.g., stability against external influences, manufacturing parameters).

## 5. Conclusions

Two experimental series (with 158 mg/L and 250 mg/L AsNa_2_HO_4_ as pre-treatment) of an arsenic-stressed duckweed bioassay presented results that were similar regarding the effect direction of relative growth rates (no effect in the first time period, growth increase in the second time period) of plants treated with potentised As_2_O_3_ compared to control groups, but had a smaller effect size compared to the original study of 2010 [13]. In all series, systematic negative control experiments indicated a stable test system. Additionally, in all series, duckweed treated with potentised As_2_O_3_ (potency levels between 17x and 33x) showed a trend of increased relative growth rates in the late time period compared to water controls. The differences were statistically significant in numerically pooled treatment and numerically pooled control groups in series 2 (250 mg/L, 16:8 h light, *p* = 0.04) and in the original series (158 mg/L, 24:0 h light, *p* < 0.001). The change of light regime (24:0 h in 2010, and 16:8 h light in the present experiments) may have had an influence on the effect size of potentised As_2_O_3_ in this bioassay. Further experiments are needed to confirm and characterise the relationship between the light regime and the effect size of potentised preparations on duckweed. 

Preparations produced according to the European Pharmacopeia monograph 2371 and 1038 (*praeparationes homoeopathicae*) repeatedly showed effects on the relative growth rate of arsenic-stressed plants in this test system. The results obtained yield empirical evidence for specific effects of potentised preparations, and call for further research into a possible underlying mode of action. 

## Figures and Tables

**Table 1:** Overview of experimental series concerning location, time and general setting of single experiments; * 10 data points were removed from SNC for alignment according to verum experiments. Sample size refers to the number of beakers with *Lemna gibba* L. No. non-stressed = number of beakers with *Lemna gibba* L. that were not pre-treated with 158 or 250 mg/mL AsNa_2_HO_4_. Chamber 0: see (Jäger et al. 2010 [13]); chamber 1 and 2: see Figure 1; differences between ImageJ analysis software “imageJ A” and “imageJ B” see Section 4.3.2 and Supplement A.

Series	Arsenic Concentration [mg/L]	Experiment	Corresponding Verum Experiment	Light Cycle (h Light:h Darkness)	Location	Chamber	Start of Experiment	Early Time Period [d]	Late Time Period [d]	Sample Size Control/Treatment	No. Non Stressed	Image Analysis Software	Starting *Ars alb* Potency	Used Potency Levels
1	158	Verum 6		16:08	Freiburg	Chamber 1	11 March 2017	0–3	3–9	40/40	4	imageJ A	5x dilution	17x, 18x, 20–23x, 28x, 30x, 33x
		Verum 7		16:08	Freiburg	Chamber 1	1 April 2017	0–3	3–9	40/40	4	imageJ A	5x dilution	17x, 18x, 20–23x, 28x, 30x, 33x
		Verum 8		16:08	Arlesheim	Chamber 1	13 February 2018	0–3	3–9	30/40	3	imageJ A	5x dilution	17x, 18x, 20–23x, 28x, 30x, 33x
		Verum 9		16:08	Arlesheim	Chamber 1	9 March 2018	0–3	3–9	30/40	4	imageJ A	5x dilution	17x, 18x, 20–23x, 28x, 30x, 33x
		Verum 10		16:08	Arlesheim	Chamber 2	11 May 2018	0–3	3–9	30/40	4	imageJ A	5x dilution	17x, 18x, 20–23x, 28x, 30x, 33x
		SNC 6	Verum 6	16:08	Freiburg	Chamber 1	2 July 2016	0–3	3–9	40/40	2	imageJ A	5x dilution	17x, 18x, 20–23x, 28x, 30x, 33x
		SNC 7	Verum 7	16:08	Freiburg	Chamber 1	15 April 2017	0–3	3–9	40/40	4	imageJ A	5x dilution	17x, 18x, 20–23x, 28x, 30x, 33x
		SNC 8 *	Verum 8	16:08	Arlesheim	Chamber 1	20 October 2017	0–3	3–9	30/40	5	imageJ A	5x dilution	17x, 18x, 20–23x, 28x, 30x, 33x
		SNC 9 *	Verum 9	16:08	Arlesheim	Chamber 1	1 December 2017	0–3	3–9	30/40	4	imageJ A	5x dilution	17x, 18x, 20–23x, 28x, 30x, 33x
		SNC 10	Verum 10	16:08	Arlesheim	Chamber 1	12 January 2019	0–3	3–9	30/40	4	imageJ A	5x dilution	17x, 18x, 20–23x, 28x, 30x, 33x
2	250	Verum 11		16:08	Arlesheim	Chamber 1	20 July 2018	0–3	3–9	30/40	5	imageJ B	5x dilution	17x, 18x, 20–23x, 28x, 30x, 33x
		Verum 12		16:08	Arlesheim	Chamber 1	7 September 2018	0–3	3–9	30/40	5	imageJ B	5x dilution	17x, 18x, 20–23x, 28x, 30x, 33x
		Verum 13		16:08	Arlesheim	Chamber 1	7 December 2018	0–3	3–9	30/40	5	imageJ B	5x dilution	17x, 18x, 20–23x, 28x, 30x, 33x
		Verum 14		16:08	Arlesheim	Chamber 2	23 May 2019	0–3	3–9	40/40	5	imageJ B	5x dilution	17x, 18x, 20–23x, 28x, 30x, 33x
		Verum 15		16:08	Arlesheim	Chamber 2	1 August 2019	0–3	3–9	40/40	5	imageJ B	5x dilution	17x, 18x, 20–23x, 28x, 30x, 33x
		SNC 11 *	Verum 11	16:08	Arlesheim	Chamber 2	9 May 2019	0–3	3–9	30/40	4	imageJ B	5x dilution	17x, 18x, 20–23x, 28x, 30x, 33x
		SNC 12 *	Verum 12	16:08	Arlesheim	Chamber 2	18 July 2019	0–3	3–9	30/40	4	imageJ B	5x dilution	17x, 18x, 20–23x, 28x, 30x, 33x
		SNC 13 *	Verum 13	16:08	Arlesheim	Chamber 2	7 November 2019	0–3	3–9	30/40	4	imageJ B	5x dilution	17x, 18x, 20–23x, 28x, 30x, 33x
		SNC 14	Verum 14	16:08	Arlesheim	Chamber 2	21 Nevember 2019	0–3	3–9	40/40	5	imageJ B	5x dilution	17x, 18x, 20–23x, 28x, 30x, 33x
		SNC 15	Verum 15	16:08	Arlesheim	Chamber 2	5 December 2019	0–3	3–9	40/40	5	imageJ B	5x dilution	17x, 18x, 20–23x, 28x, 30x, 33x
0	158	Verum 1		24:00	Frick	Chamber 0	25 March 2009	0–2	2–6	45/45	5	Scanalyzer	5x trituration	17x, 18x, 20–24x, 28x, 30x, 33x
(orig.		Verum 2		24:00	Frick	Chamber 0	3 June 2009	0–2	2–6	45/45	5	Scanalyzer	5x trituration	17x, 18x, 20–24x, 28x, 30x, 33x
series)		Verum 3		24:00	Frick	Chamber 0	29 July 2009	0–2	2–6	45/45	5	Scanalyzer	5x trituration	17x, 18x, 20–24x, 28x, 30x, 33x
		Verum 4		24:00	Frick	Chamber 0	12 August 2009	0–2	2–6	45/45	5	Scanalyzer	5x trituration	17x, 18x, 20–24x, 28x, 30x, 33x
		Verum 5		24:00	Frick	Chamber 0	28 August 2009	0–2	2–6	45/45	5	Scanalyzer	5x trituration	17x, 18x, 20–24x, 28x, 30x, 33x
		SNC 1	Verum 1	24:00	Frick	Chamber 0	20 January 2009	0–2	2–6	45/45	5	Scanalyzer	5x trituration	17x, 18x, 20–24x, 28x, 30x, 33x
		SNC 2	Verum 2	24:00	Frick	Chamber 0	22 April 2009	0–2	2–6	45/45	5	Scanalyzer	5x trituration	17x, 18x, 20–24x, 28x, 30x, 33x
		SNC 3	Verum 3	24:00	Frick	Chamber 0	10 June 2009	0–2	2–6	45/45	5	Scanalyzer	5x trituration	17x, 18x, 20–24x, 28x, 30x, 33x
		SNC 4	Verum 4	24:00	Frick	Chamber 0	8 July 2009	0–2	2–6	45/45	5	Scanalyzer	5x trituration	17x, 18x, 20–24x, 28x, 30x, 33x
		SNC 5	Verum 5	24:00	Frick	Chamber 0	2 September 2009	0–2	2–6	45/45	5	Scanalyzer	5x trituration	17x, 18x, 20–24x, 28x, 30x, 33x

**Table 2:** Coefficient of variance (CV) for relative growth rates in the systematic negative control experiments of both experimental series, representing variance between randomisation groups of each experiment (CV = SD × 100/mean, based on mean values of randomisation groups).

CV Series 1	Early Time Period (Day 0–3)	Late Time Period (Day 3–9)	CV Series 2	Early Time Period (Day 0–3)	Late Time Period (Day 3–9)
SNC 1	3.00	2.27	SNC 1	1.20	1.38
SNC 2	1.35	1.25	SNC 2	1.66	1.24
SNC 3	2.02	0.83	SNC 3	1.69	0.97
SNC 4	1.96	0.79	SNC 4	1.65	0.86
SNC 5	0.90	0.73	SNC 5	2.59	1.04
SNC 1–5	1.78	1.14	SNC 1–5	1.75	1.10

**Table 3:** Statistical analysis of experimental series 1. Results of two-way ANOVA of series 1 for numerically pooled treatment (As_2_O_3_ 17x–18x, 21x–23x, 28x, 30x, and 33x) and numerically pooled control (unsuccussed and succussed water) groups for n = 5 independent experiments in the early (day 0–3) and late time period (day 3–9). df—degree of freedom. Significant values are printed bold.

Series 1 Early Time Period (Day 0–3)	df	Sum of Squares	F Ratio	*p* Value
Treatment group	1	0.00000023	0.0013	0.9712
Experiment number	4	0.08786236	125.4573	**<0.0001**
Interaction	4	0.00027976	0.3995	0.809
**Series 1** **Late Time Period (Day 3–9)**	**df**	**Sum of Squares**	**F Ratio**	***p*** **Value**
Treatment group	1	0.00019524	2.7635	0.0973
Experiment number	4	0.06368114	225.3325	**<0.0001**
Interaction	4	0.0004133	1.4624	0.2131

**Table 4:** Statistical analysis of experimental series 1. Results of a two-way ANOVA of series 1 for single treatment (As_2_O_3_ 17x–18x, 21x–23x, 28x, 30x, and 33x) and control (unsuccussed and succussed water) groups for n = 5 experiments in early (day 0–3) and late time period (day 3–9). df—degree of freedom. Significant values are printed bold.

Series 1 Early Time Period (Day 0–3)	df	Sum of Squares	F Ratio	*p* Value
Treatment group	9	0.00057831	0.3431	0.9599
Experiment number	4	0.0612927	81.8193	**<0.0001**
Interaction	36	0.00275917	0.4092	0.9991
**Series 1** **Late Time Period** **(Day 3–9)**	**df**	**Sum of Squares**	**F Ratio**	***p*** **Value**
Treatment group	9	0.00029427	0.4437	0.9106
Experiment number	4	0.04412396	149.7021	**<0.0001**
Interaction	36	0.00217615	0.8204	0.7605

**Table 5:** Statistical analysis of experimental series 2. Results of two-way ANOVA of series 2 for numerically pooled treatment groups (As_2_O_3_ 17x–18x, 21x–23x, 28x, 30x, and 33x) and numerically pooled control groups (unsuccussed and succussed water) for n = 5 independent experiments in the early (day 0–3) and late time period (day 3–9). df—degree of freedom. Significant values are printed bold.

Series 2 Early Time Period (Day 0–3)	df	Sum of Squares	F Ratio	*p* Value
Treatment group	1	0.00000771	0.0705	0.7908
Experiment number	4	0.21284402	486.3185	**<0.0001**
Interaction	4	0.0005176	1.1826	0.3181
**Series 2** **Late Time Period** **(Day 3–9)**	**df**	**Sum of Squares**	**F Ratio**	***p*** **Value**
Treatment group	1	0.00029398	4.0814	**0.0441**
Experiment number	4	0.03503934	121.617	**<0.0001**
Interaction	4	0.0002879	0.9993	0.4079

**Table 6:** Statistical analysis of experimental series 2. Results of a two-way ANOVA of series 2 for single treatment (As_2_O_3_ 17x–18x, 21x–23x, 28x, 30x, and 33x) and control (unsuccussed and succussed water) groups, analysed over n = 5 experiments in early (day 0–3) and late time period (day 3–9). df—degree of freedom. Significant values are printed bold.

Series 2 Early Time Period (Day 0–3)	df	Sum of Squares	F Ratio	*p* Value
Treatment group	9	0.00063312	0.6484	0.7553
Experiment number	4	0.16981641	391.2808	**<0.0001**
Interaction	36	0.00454901	1.1646	0.245
**Series 2** **Late Time Period** **(Day 3–9)**	**df**	**Sum of Squares**	**F Ratio**	***p*** **Value**
Treatment group	9	0.00073737	1.1146	0.3517
Experiment number	4	0.02844692	96.7118	**<0.0001**
Interaction	36	0.00223863	0.8456	0.723

**Table 7:** Statistical analysis of experimental series 1 and 2. Results of three-way ANOVA for numerically pooled treatment (As_2_O_3_ 17x–18x, 21x–23x, 28x, 30x, and 33x) and numerically pooled control groups (unsuccussed and succussed water) for n = 2 experimental series with n = 5 independent experiments, for the early (day 0–3) and late time period (day 3–9). df—degree of freedom. * = interaction between independent ANOVA factors. Significant values are printed bold.

Pool Series 1 + 2 Early Time Period (Day 0–3)	df	Sum of Squares	F Ratio	*p* Value
Treatment group (tg)	1	0.00000529	0.0372	0.8471
Experiment number (en)	4	0.14827807	260.594	**<0.0001**
Experimental series (es)	1	0.01723593	121.1664	**<0.0001**
tg * en	4	0.00063099	1.1089	0.3511
tg * es	1	0.00000264	0.0186	0.8916
en * es	4	0.13905187	244.3792	**<0.0001**
tg * en * es	4	0.00015998	0.2812	0.8902
**Pool Series 1 + 2** **Late Time Period** **(Day 3–9)**	**df**	**Sum of Squares**	**F Ratio**	***p*** **Value**
Treatment group (tg)	1	0.00048419	6.787	**0.0094**
Experiment number (en)	4	0.05417827	189.8587	**<0.0001**
Experimental series (es)	1	0.01532783	214.8553	**<0.0001**
tg * en	4	0.00053627	1.8793	0.1122
tg * es	1	0.00000503	0.0706	0.7906
en * es	4	0.0399193	139.8905	**<0.0001**
tg * en * es	4	0.00015089	0.5288	0.7146

**Table 8:** Statistical analysis of series 1 and 2. Results of a three-way ANOVA for single treatment (As_2_O_3_ 17x–18x, 21x–23x, 28x, 30x, and 33x) and control (unsuccussed and succussed water) groups for n = 2 experimental series with n = 5 independent experiments, for the early (day 0–3) and late time period (day 3–9). df—degree of freedom. * = interaction between independent ANOVA factors. Significant values are printed bold.

Pool Series 1 + 2 Early Time Period (Day 0–3)	df	Sum of Squares	F Ratio	*p* Value
Treatment group (tg)	9	0.00029496	0.2216	0.9914
Experiment number (en)	4	0.11806456	199.581	**<0.0001**
Experimental series (es)	1	0.01398425	94.5581	**<0.0001**
tg * en	36	0.00418589	0.7862	0.8117
tg * es	9	0.00091647	0.6885	0.7197
en * es	4	0.11179632	188.9849	**<0.0001**
tg * en * es	36	0.00316782	0.595	0.972
**Pool Series 1 + 2** **Late Time Period** **(Day 3–9)**	**df**	**Sum of Squares**	**F Ratio**	***p*** **Value**
Treatment group (tg)	9	0.00078236	1.1809	0.3042
Experiment number (en)	4	0.04162298	141.3617	**<0.0001**
Experimental series (es)	1	0.01252126	170.1009	**<0.0001**
tg * en	36	0.00246072	0.9286	0.5908
tg * es	9	0.00024958	0.3767	0.9463
en * es	4	0.03053381	103.7002	**<0.0001**
tg * en * es	36	0.0019505	0.736	0.8718

## Data Availability

The dataset of this study is available upon request to the corresponding author.

## Supplementary Materials

The following are available online at https://www.mdpi.com/article/10.3390/biomedicines10030552/s1, Supplement A: Macros for image analysis using the program “imageJ”. Supplement B: Table two-way ANOVA for SNC experiments for early and late time periods in series 1 and 2.
