# Supplementary material for: Critical Evaluation of Specific Efficacy of Preparations Produced According to European Pharmacopeia Monograph 2371
Source: Biomedicines. 2022 Feb 25;10(3):552. doi: 10.3390/biomedicines10030552 (PMC8944999; doi:10.3390/biomedicines10030552)
Supplement: Supplementary file 1 [file biomedicines-10-00552-s001.zip › Supplement A.pdf]

biomedicines
MDPI

Supplement A: Image-Analysis: ImageJ Macros 1 and 2

Macro 1

```
//Macro 1 for Area-Detection of Duckweed from digital photos, saving mask for comprehensability
//Additional Program is necessary: IHC_Toolbox (Shu, J. , G. Qiu, M. Ilyas, and P. Kaye. 2010. Biomarker Detection in Whole Slide Imaging based on Statistical Color Models. MIDAS Journal)
//First step necessary: Create a File with IHC-Toolbox defining the used green value of the duckweed fronds
//For this, use the "Training" button on a sample of pictures (app. 10 pictures) from multiple days (day 0,3 and 9) and if detection seems optimal, save settings
//now Macro 1 can be started
//Use this file for detection at the step "waiForUser("IHC_Toolbox...)" and correct by hand, if necessary
var input = "...";
var output = "...";
	macro "Set Directory to save Results" {
	output = getDirectory("Choose Destination Directory");
	print(output);
}
	macro "Wasserlinsen Bildbearbeitung" {
	input = getDirectory("Choose the Directory where the file is");
	list = getFileList(input);
	for(i = 0; i < list.length; i++) {
		Lemna(list[i]);
	}
}
	function Lemna(img_filename) {
		fullpath_image = input + img_filename;
		open(fullpath_image);
		sourceID = getImageID();
		img_title = getTitle();

		run("Duplicate...", " ");
		waitForUser("IHC_Toolbox for green filter and then root editing, then press ok or enter");
		run("8-bit");
		dest_outlinename = img_title+"_outline.tif";
		fullpath = output + dest_outlinename;
		saveAs("tiff", fullpath);
		setThreshold(0, 254);
		run("Convert to Mask");
		run("Measure");
		selectImage(sourceID);
		close("*");
	dest_filename = img_title+"_measure.xls";
	fullpath = output + dest_filename;
```

```
        print(fullpath);
        saveAs("Measurements", fullpath);
    }
```

Macro 2

//Macro 2 for Area-Detection of Duckweed from digital photos if duckweed has chlorotic (white) parts at border of frond, saving mask for comprehensability

//No additional program is necessary

//at step "waitForUser" implement wand-Tool and ROI-Manager for selection of all fronds, correct by hand if necessary

```
    var input = "...";
    var output = "...";
        macro "Set Directory to save Results" {
        output = getDirectory("Choose Destination Directory");
        print(output);
    }
        macro "Wasserlinsen Bildbearbeitung mit chlorotischem Rand" {
        input = getDirectory("Choose the Directory where the file is");
        list = getFileList(input);
        for(i = 0; i  <  list.length; i++) {
            Lemna(list[i]);
    }
    }
        function Lemna(img_filename) {
            fullpath_image = input + img_filename;
            open(fullpath_image);
            sourceID = getImageID();
            img_title = getTitle();
            run("Duplicate...", " ");
            waitForUser("use wand and ROI Manager to select fronds. Mark all ROI with OR(COMBINE)");
            run("Create Mask");
            dest_outlinename = img_title+"_outline.tif";
            fullpath = output + dest_outlinename;
            saveAs("tiff", fullpath);
            run("Measure");
            selectImage(sourceID);
            close("*");

        dest_filename = img_title+"_measure.xls";
        fullpath = output + dest_filename;
        print(fullpath);
        saveAs("Measurements", fullpath);
    }
```
