# Supplementary material for: Critical Evaluation of Specific Efficacy of Preparations Produced According to European Pharmacopeia Monograph 2371
Source: Biomedicines. 2022 Feb 25;10(3):552. doi: 10.3390/biomedicines10030552 (PMC8944999; doi:10.3390/biomedicines10030552)
Supplement: Supplementary file 1 [file biomedicines-10-00552-s001.zip › SupplementB.pdf]

biomedicines

MDPI

Supplement B Statistical analysis of the systematic negative control experiments for series 1 and 2.

Table A. Statistical analysis of the systematic negative control experiments. Results of a two-way ANOVA with the independent factors experiment number (n=5) and pseudo-treatment (n=2), and the dependent (outcome) variable area-related relative growth-rate for series 1 and series 2, and for early (day 0–3) and late time period (day 3–9).

**SNC Series 1 Early time period (day 0-3)**	**df**	**Sum of squares**	**F ratio**	**p value**
Treatment group	1	0.00000027	0.0016	0.9684
Experiment number	4	0.39488115	575.3402	**<0.0001**
Interaction	4	0.00014737	0.2147	0.9302

**SNC Series 2 Early time period (day 0-3)**	**df**	**Sum of squares**	**F ratio**	**p value**
Treatment group	1	0.00000341	0.0273	0.8688
Experiment number	4	0.0237487	47.5884	**<0.0001**
Interaction	4	0.00042096	0.8435	0.4983

**SNC Series 1 Late time period (day 3-9)**	**df**	**Sum of squares**	**F ratio**	**p value**
Treatment group	1	0.00001871	0.1939	0.66
Experiment number	4	0.18253569	472.7993	**<0.0001**
Interaction	4	0.00013553	0.351	0.8433

**SNC Series 2 Late time period (day 3-9)**	**df**	**Sum of squares**	**F ratio**	**p value**
Treatment group	1	0.00001236	0.1882	0.6647
Experiment number	4	0.01409492	53.6795	**<0.0001**
Interaction	4	0.00027277	1.0388	0.387

Table B. Statistical analysis of the systematic negative control experiments. Results of a three-way ANOVA with the independent factors experimental series (n=2), experiment number (n=5) and pseudo-treatment (n=2), and the dependent (outcome) variable area-related relative growth-rate for day 0–3 and day 3–9. df – degree of freedom

**SNC Pool Series 1+2 Early time period (day 0-3)**	**df**	**Sum of squares**	**F ratio**	**p value**
Treatment group (tg)	1	0.00000088	0.0059	0.9386

Experiment number (en)	4	0.13174687	222.2848	**<0.0001**
Experimental series (es)	1	0.0613557	414.0801	**<0.0001**
Tg*en	4	0.00031784	0.5363	0.7091
Tg*es	1	0.0000028	0.0189	0.8907
En*es	4	0.27195529	458.846	**<0.0001**
Tg*en*es	4	0.00026107	0.4405	0.7794

**SNC Pool Series 1+2 Late time period (day 3-9)**	**df**	**Sum of squares**	**F ratio**	**p value**
Treatment group (tg)	1	0.00003074	0.3791	0.5383
Experiment number (en)	4	0.10719041	330.5031	**<0.0001**
Experimental series (es)	1	0.01100546	135.7338	**<0.0001**
Tg*en	4	0.00034938	1.0773	0.3667
Tg*es	1	0.00000033	0.0041	0.9493
En*es	4	0.08808902	271.6073	**<0.0001**
Tg*en*es	4	0.00007078	0.2182	0.9283
